# Exercise in obese pregnant women: The role of social factors, lifestyle and pregnancy symptoms

**DOI:** 10.1186/1471-2393-11-4

**Published:** 2011-01-12

**Authors:** Katie F Foxcroft¹, Ingrid J Rowlands, Nuala M Byrne, H David McIntyre, Leonie K Callaway

**Affiliations:** 1 Department of Internal Medicine and Aged Care, Royal Brisbane and Women's Hospital, (Butterfield St), Brisbane, (4029), Australia; 2 Department of Epidemiology, Queensland Institute of Medical Research and School of Population Health, University of Queensland, (Herston Rd), Brisbane, (4029), Australia; 3 School of Human Movement Studies and Institute of Health and Biomedical Innovation, Queensland University of Technology, Musk Ave, Brisbane,(4059), Australia; 4 Department of Endocrinology and Obstetric Medicine, Mater Health Services and Mater Clinical School, University of Queensland, Stanley St, Brisbane, (4101), Australia; 5 Department of Internal Medicine Services, Royal Brisbane and Women's Hospital and School of Medicine, University of Queensland, (Herston Rd) Brisbane, (4029), Australia

## Abstract

**Background:**

Physical activity may reduce the risk of adverse maternal outcomes, yet there are very few studies that have examined the correlates of exercise amongst obese women during pregnancy. We examined which relevant sociodemographic, obstetric, and health behaviour variables and pregnancy symptoms were associated with exercise in a small sample of obese pregnant women.

**Methods:**

This was a secondary analysis using data from an exercise intervention for the prevention of gestational diabetes in obese pregnant women. Using the Pregnancy Physical Activity Questionnaire (PPAQ), 50 obese pregnant women were classified as "Exercisers" if they achieved ≥900 kcal/wk of exercise and "Non-Exercisers" if they did not meet this criterion. Analyses examined which relevant variables were associated with exercise status at 12, 20, 28 and 36 weeks gestation.

**Results:**

Obese pregnant women with a history of miscarriage; who had children living at home; who had a lower pre-pregnancy weight; reported no nausea and vomiting; and who had no lower back pain, were those women who were most likely to have exercised in early pregnancy. Exercise in late pregnancy was most common among tertiary educated women.

**Conclusions:**

Offering greater support to women from disadvantaged backgrounds and closely monitoring women who report persistent nausea and vomiting or lower back pain in early pregnancy may be important. The findings may be particularly useful for other interventions aimed at reducing or controlling weight gain in obese pregnant women.

## Background

Physical activity during pregnancy is important for women's general health and may reduce the risk of adverse maternal, fetal and neonatal outcomes. Current recommendations advise pregnant women without medical or obstetric complications to aim for 30 minutes of physical activity on most days of the week [1]. Randomised controlled trials have shown that the uptake of regular exercise among sedentary pregnant women has significant benefits for women in pregnancy. Specifically, women who participated in three hours of weekly vigorous exercise in pregnancy reported greater satisfaction with their physical stamina, energy levels, appearance and general health than sedentary pregnant women [2]. In another study of pregnant women who were overweight, participation in three hours of aerobic exercise per week was associated with higher fitness levels as demonstrated by increased oxygen uptake, than overweight women who remained sedentary [3]. Exercise also appears to have benefits for neonates, with the uptake of moderate-intensity exercise in pregnancy being associated with normal fetal growth [4]. However, some women may have difficulty meeting current recommendations, or participating in physical activity altogether during pregnancy because of health and psychosocial factors.

The correlates of physical activity among women during pregnancy have been examined in only a few studies. Sociodemographic variables such as education and income [5-8] have been positively associated with physical activity in pregnancy whereas a negative association has been found for age [7,9]. Women who have children at home and an unfavourable pregnancy history [9] are less likely to be physically active during pregnancy. Although there is limited evidence, psychosocial variables such as employment during pregnancy and lack of childcare [10] have also been identified as correlates of physical activity in pregnancy [11]. However, overall the evidence tends to be conflicting with several studies finding no relationship between these variables and women's physical activity levels during pregnancy.

Women's physical health and health behaviours before and during pregnancy may be important predictors of physical activity during pregnancy. Women who have a high pre-pregnancy body mass index (BMI) [11] or who smoke [6,7] are less likely to be physically active in pregnancy. On the other hand, pre-pregnancy physical activity has been associated with remaining physically active during pregnancy [10]. Remaining active during pregnancy may be beneficial for women's well-being, with one study showing that exercise during the first trimester of pregnancy was related to reduced reporting of nausea and vomiting in the 2^nd ^trimester of pregnancy[12].

Physical symptoms are common and normal in pregnancy, but they may deter or prevent some women from exercising during pregnancy. For example, almost 80% of pregnant women experience nausea and vomiting [12] in their first trimester. Although this generally resolves by the 12th week of pregnancy, around 40% of women report nausea and vomiting into their second trimester and some women are affected for the entire pregnancy. Further, back pain during pregnancy affects anywhere between 24 and 90% of women and may interfere with women's ability to exercise [13,14]. While pregnancy symptoms may have a large impact on women's wellbeing, there is evidence to suggest that exercise may improve women's symptoms[8].The correlates of exercise during pregnancy among women who are obese have not been examined. Women who are obese are at the greatest risk for pregnancy complications [15] and weight retention in the longer term [16], and thus it seems important to examine whether there are factors that are common to obese pregnant women who do exercise. This study examines the correlates of exercise in a small sample of pregnant women who were identified as achieving, or not achieving adequate exercise-specific energy expenditure requirements throughout their pregnancy. We expected that the relevant sociodemographic, obstetric and health behaviour variables, and pregnancy symptoms, would be associated with exercise during pregnancy in this group of women. This information would be useful for informing lifestyle interventions that aim to reduce or control weight gain among obese pregnant women.

## Methods

### Participants

This is a secondary analysis of a study of 50 women who were recruited as part of a randomised controlled trial (RCT) examining the feasibility of an individualised exercise program for obese women during pregnancy. Full details of study design and participant recruitment have been reported previously [17]. Women receiving antenatal care and delivering at the Royal Brisbane and Women's Hospital (RBWH) in Queensland, Australia, were recruited from the hospital's maternity outpatient clinic between 12 and 14 weeks gestation. Ethics clearance was obtained for the study from the Royal Brisbane and Women's Hospital (RBWH) Human Research Ethics Committee. The RCT study is registered with the Australian Clinical Trials Registry (ACTRN012606000271505). Women were included in the study if they were: aged 18-45; had a BMI of 30 kg.m^-2 ^or greater; were willing to participate in an exercise program; and able to provide informed consent. Exclusion criteria included: non-English speaking; contraindication or inability to exercise; medical or obstetric contraindication to exercise including hemodynamically significant heart disease; restrictive lung disease; incompetent cervix (cerclage); multiple gestation; severe anaemia; chronic bronchitis; type 1 diabetes; orthopaedic limitations; poorly controlled seizure disorder; poorly controlled hyperthyroidism; heavy smoker.

*Pre-intervention Stage*. All eligible women were invited to attend a single early group education session at around 12 week's gestation. Women received written information on exercise[1], nutrition [18] and weight gain during pregnancy[19]. The women were subsequently invited to attend a face-to-face interview with a research midwife. Interviews collected information on demographics and physical and mental health and health behaviours.

### Intervention

Women randomized to the intervention received a) an individualised exercise plan b) regular exercise advice and c) paper-based diaries for self-monitoring. A face-to-face interview at ≈12 weeks with a physiotherapist, who had expertise in pregnancy management and exercise physiology, was conducted to develop women's individualized exercise plans; to assess readiness for change, and encourage goal setting. Women were reviewed every 4 weeks by physiotherapists, with phone calls between visits to assess their adherence to the program. Women who were not meeting exercise targets had additional face-to-face support, with identification of barriers and modification of the exercise plan.

Both the intervention and control groups were followed up at 12, 20, 28 and 36 weeks by a research midwife who recorded their weight, pregnancy symptoms and administered the PPAQ.

### Energy Expenditure

We examined the correlates of energy expenditure, which are expressed as kilocalories per week (kcal) in this paper. Energy expenditure was derived from the Pregnancy Physical Activity Questionnaire (PPAQ). Data was collected at 12, 20, 28 and 36 weeks gestation.

#### Pregnancy physical activity questionnaire (PPAQ; [20])

This is a self-report instrument which measures the time spent participating in 32 activities including household/care giving, occupational, sports/exercise, transportation, and inactivity. The PPAQ is reliable and valid measure of exercise during pregnancy. Specifically, the intraclass correlation coefficient for the sports and exercise activity subscale was 0.83, and scores for the sports and exercise subscale correlate moderately with actigraph data [20].

From the PPAQ, we extracted data for sports and exercise activities only. The types of sports and exercise activities assessed in the PPAQ include walking, jogging, prenatal exercise classes, swimming and dancing. To calculate weekly energy expenditure using the PPAQ, the duration of time spent in these exercise activities was multiplied by specific intensities (i.e. MET values) and scores are expressed as MET-hours per week.

In order take into account the women's weight, which can greatly affect energy expenditure, weekly kilocalories (kcals) expended by the women during exercise was calculated. Weekly kcals at each time point were derived by multiplying MET-hours per week by weight (kg). Because the data was severely skewed, it was converted into categorical outcome variables at each time point. Because there are no recommended cut-offs, we chose the cut-point of 900 kcal/wk based the results of prior exercise intervention delivered to non-pregnant, obese individuals [21]. Thus, at each time point, women who achieved < 900 kcal/wk were classified as "Non-exercisers" and women who achieved >900 kcal/wk were classified as "Exercisers".

### Predictor Variables

#### Background information

A semi-structured interview was used to collect information on maternal sociodemographic, obstetric, and health and health behaviour characteristics. The woman's height was measured at this interview and using self-reported pre-pregnancy weight, pre-pregnancy BMI was calculated.

#### Pregnancy symptoms

Women were asked to describe their pregnancy symptoms at 12, 20, 28 and 36 weeks. Based on women's qualitative descriptions of their symptoms at each time point, separate dichotomous variables were created for nausea/vomiting and fatigue. For each symptom, women who reported having had the symptom were coded as "*Yes*", and women without this symptom were coded as "*No*".

The number of symptoms reported by women was also used to create variables showing the total number of symptoms reported at each time point. These variables were categorised as: *0-1*; *2-3*; *4 or more*.

#### Low back pain

The Roland-Morris Disability questionnaire (RDQ-24) is a self-report questionnaire assessing the level of physical disability resulting from low back pain. The questionnaire contains 24 statements describing symptoms of low back pain, and individuals are asked tick only those statements which apply to them on the day of completion. Total scores range from 0 to 24, with higher scores indicating greater disability. The RDQ-24 has been shown to be a reliable measure of low back pain that has been validated in a number of populations and countries. Internal consistency for the scale is good with Cronbach's alpha ranging from 0.84 to 0.93[22].

### Statistical Analysis

All analyses were conducted using STATA version 10.0 (StataCorp, Texas, USA). Univariate differences between the groups on the sociodemographic, obstetric, physical and mental health and health-related variables, and pregnancy symptoms, were examined using chi-square tests for independence and Fisher's exact tests for categorical variables, and unpaired t-tests for continuous variables. Women were included in the analyses at each time point if they had data on the PPAQ.

## Results

### Participant Characteristics

The mean age of the sample (n = 50) was 30 ±5 yrs and slightly more than half of women were married and had at least one child (Table 1). Most women (74%) reported being in either part-time or full-time employment at 12 weeks gestation and 30% were tertiary educated. Almost one fifth of women had a history of miscarriage and 26% had had a previous caesarean section. The median pre-pregnancy weight was 90 kg and 36% of women had a BMI greater than 35 kg.m^-2^, but smoking rates were relatively low (6%). At 12 weeks gestation, nausea and fatigue affected 78% and 54% of women, respectively. Back pain affected 6% of women at 12 weeks and increased to 30.5% at 36 weeks gestation. Characteristics of the women according to their group allocation in the randomised control trial have been published elsewhere [17].

**Table1:** Sociodemographic, health, obstetric, and health behaviour predictors for women classified, Exercisers and Non-exercisers during pregnancy

	**12 weeks**^**4**^		**20 weeks**^**4**^		**28 weeks**^**4**^		**36 weeks**^**4**^	
VARIABLE	*n *= 33	*n *= 17	*n *= 18	*n *= 24	*n *= 17	*n *= 24	*n *= 21	*n *= 15
	Non-Exercisers	Exercisers	Non-Exercisers	Exercisers	Non-Exercisers	Exercisers	Non-Exercisers	Exercisers
**RCT study group** *Control Intervention*	18 (55%) 15 (45%)	7 (41%) 10 (59%)	10 (63%) 6 (38%)	9 (38%) 15 (63%)	11 (65%) 6 (35%)	8 (33%) 16 (67%)	11 (55%) 9 (45%)	5 (33%) 10 (67%)
***p***	0.37^3^		0.12^3^		**0.047**^3^		0.20^3^	
**Age M (SD)**	30.0 (5.7)	30.5 (4.7)	29.6 (5.4)	30.3 (5.2)	31.9 (4.5)	29.1 (5.4)	29.4 (5.4)	32.1 (4.4)
***p***	0.39		0.70		**0.09**		0.12	
**Tertiary**	10 (30%)	5 (30%)	4 (22%)	11 (46%)	5 (29%)	10 (42%)	4 (19%)	9 (60%)
***p***	0.95^1^		0.19^3^		0.42^1^		**0.02**^3^	
**Marital Status**								
*Married*	24 (73%)	9 (53%)	10 (56%)	17 (71%)	9 (53%)	17 (71%)	13 (62%)	10 (67%)
***p***	0.16^1^		0.31^1^		0.24^1^		0.77^1^	
**Employed**	24 (73%)	13 (76%)	16 (89%)	17 (71%)	16 (94%)	17 (81%)	18 (86%)	11 (73%)
***p***	0.77^3^		0.29^3^		0.11^3^		0.42^3^	
≥**1 child at home**	18 (55%)	12 (71%)	7 (39%)	17 (81%)	8 (47%)	16 (67%)	14 (67%)	9 (60%)
***p***	0.27^3^		**0.038**^**1**^		0.21^1^		0.68^1^	
**Previous miscarriages**	3 (9%)	6 (35%)	1 (6%)	6 (25%)	1 (6%)	6 (25%)	3 (14%)	4 (27%)
***p***	**0.047**^**1**^		0.21		0.21		0.42	
**Previous caesarean section**	8 (24%)	5 (2(%)	3 (17%)	7 (33%)	3 (18%)	7 (29%)	3 (14%)	6 (40%
***p***	0.69^3^		0.30^3^		0.48^3^		0.12^3^	
**Smoker**	3 (9%)	0(0%)	2 (11%)	1 (4%)	2 (12%)	0 (0%)	2 (10%)	0 (0%)
***p***	0.54^1^		0.57^3^		0.17^3^		0.50^3^	
**Pre-pregnancy weight**	93 (75-176)	85 (68-95)	92 (75-176)	87.5 (68-105)	93 (68-176)	87.5 (78-115)	93 (68-175)	89 (78.2-176)
***p***	**0.01**^**2**^		0.31^2^		0.48^2^		0.52^2^	
**History of mental health diagnoses**	11 (33%)	8 (47%)	7 (39%)	8 (33%)	6 (35%)	8 (33%)	9 (43%)	4 (27%)
***p***	0.34^3^		0.71^1^		0.89^1^		0.32^3^	

*Note. *^1^Chi-sqaure test. ^2^Mann-Whitney test. Data for pre-pregnancy weight is presented as: Median (range). ^3^Fisher's exact test. ^4^Numbers may not sum to total because some data missing

### Energy Expenditure

Table 1 shows the proportion of women who were classified as *Exercisers *and *Non-exercisers*. At 12 weeks gestation, the proportion of *Non-exercisers *(66%) was almost double that of *Exercisers *(34%). Therefore, more than half of the women were not achieving greater than 900 kcal of exercise per week. Although there was a marked decrease in the proportion of *Non-exercisers *at 20 (40%) and 28 (41%) weeks, the percentage of *Non-Exercisers *increased to 57% at 36 weeks. Data derived from the semi-structured interviews with the women conducted as part of the RCT showed that walking was the preferred method of exercise over the course of the trial, although a wide variety of activities were undertaken [23].

### Predictors of Exercise in Early Pregnancy

Table 1 shows the selected sociodemographic, obstetric, health and health behaviour predictors of exercise at 12 weeks gestation for women who were classified *Exercisers *and *Non-exercisers*. Pre-pregnancy weight and a history of previous miscarriages were associated with exercise status. Exercisers were more likely to have a lower pre-pregnancy weight (*z *= 2.74, *p *= 0.006) and a history of miscarriage than non-exercisers (*p *= 0.047, Fisher's exact test). The number of children living at home was also a determinant of exercise status. At 20 weeks, *Exercisers *were more likely to have at least one child living at home than *Non-exercisers *χ^2 ^(*df *= 1, *N *= 42) = 4.29,

*p *= 0.038.

### Predictors of Exercise in Late Pregnancy

Educational attainment was a significant predictor of exercise in late pregnancy (see Table 1). At 36 weeks, women who had completed tertiary education were three times more likely to be an *Exerciser *(*p *= 0.02, Fisher's exact test). At 28 weeks, women who were randomised to the intervention group in the RCT were more likely to be classified as an *Exerciser *χ^2^(*df *= 1, *N *= 41) = 3.94, *p *= 0.047, and there was also a trend towards *Exercisers *being slightly younger than the *Non-Exercisers *(*p *= 0.09, Fisher's exact test).

### Pregnancy Symptoms

Self-reported pregnancy symptoms were associated with exercise status during pregnancy (Table 2). Specifically, women classified as *Exercisers *were less likely to report nausea or vomiting at 28 weeks gestation than *Non-exercisers *(*p *= 0.01, Fisher's exact test). Differences between the *Exercisers *and *Non-exercisers *were also found for low back pain. The distributions of low back pain for the *Exercisers *and *Non-exercisers *showed that *Exercisers *were more likely to report fewer symptoms of back pain than *Non-*exercisers (*z *= 1.99, *p *= 0. 047). There were no significant differences between the groups for fatigue and poor sleep at any point during pregnancy.

**Table 2:** Number of pregnancy symptoms: nausea, vomiting, fatigue, low back pain during pregnancy in PPAQ Exercisers versus PPAQ Non-exercisers

	**12 weeks**^**4**^		**20 weeks**^**4**^		**28 weeks**^**4**^		**36 weeks**^**4**^	
VARIABLE	*n *= 33	*n *= 17	*n *= 18	*n *= 24	*n *= 17	*n *= 24	*n *= 21	*n *= 15
No. of Symptoms	Non-exercisers	Exercisers	Non-Exercisers	Exercisers	Non-Exercisers	Exercisers	Non-Exercisers	Exercisers
0 - 1	9 (27%)	4 (24%)	5 (28%)	14 (58%)	4 (24%)	10 (42%)	2 (10%)	3 (20%)
2 - 3	17 (52%)	11 (65%)	10 (56%)	8 (33%)	7 (41%)	12 (50%)	14 (70%)	9 (60%)
4 or more	7 (21%)	2 (12%)	3 (17%)	2 (8%)	6 (35%)	2 (8%)	4 (20%)	3 (20%)
***p***	0.72^3^		0.15^3^		**0.09**^**3**^		0.88^3^	
**Nausea & Vomiting**	3 (14%)	6 (37%)	8 (44%)	3(13%)	7(41%)	1 (4%)	1(5%)	2 (13%)
***p***	0.136^3^		**0.033**^**3**^		**0.005**^**3**^		0.565^3^	
**Fatigue**	17 (52%)	11(65%)	3 (17%)	9 (38%)	7 (41%)	3 (13%)	3 (15%)	2 (13%)
***p***	0.37^1^		0.18^3^		0.63^3^		1.00^3^	
	***n *= 32**	***n *= 16**	***n *= 14**	***n *= 23**	***n *= 15**	***n *= 23**	***n *= 21**	***n *= 14**
**Back Pain**	1 (0-15)	0 (0-7)	3 (0-14)	0 (0-10)	1 (0-8)	2 (0-18)	3 (0-19)	2 (0-24)
***p***	**0.006**^**2**^		**0.03**^**2**^		0.30^**2**^		0.25^**2**^	

*Note. *^1^Chi-sqaure test. ^2^Mann-Whitney test. Data for back pain is presented as: Median (range). ^3^Fisher's exact test. ^4 ^Numbers may not sum to total because some data missing.

## Discussion

The aim of the study was to examine the correlates of exercise among obese women in early and late pregnancy. Although relevant sociodemographic, health behaviour variables and pregnancy symptoms were associated with exercise status in this study, the only obstetric variable to show an association with exercise status was previous miscarriages. Women who had a history of miscarriage were more likely to be *Exercisers *very early in pregnancy. This is likely to reflect the fact that many pregnant women who have a history of pregnancy loss are anxious about future loss and may subsequently adopt healthier lifestyles in an attempt to prevent future miscarriages [24,25].

Sociodemographic variables, including the number of children living at home and education were associated with exercise status in this study. We found that women with at least one child living at home were more likely to be classified as *Exercisers*, which is contrary to other evidence showing that women with children at home are less likely to be physically active [6,9,10]. It is possible that the women in our study with children were also not currently working, or had children who were older and in school. This may have allowed the women to have more time to exercise accounting for our findings. We found that education was a predictor of exercise status in late pregnancy. Consistent with previous findings [6,8,9,26], women classified as *Exercisers *were more likely to have completed tertiary education than *Non-exercisers*. Our findings are not unexpected considering that the link between obesity and low socio-economic status has been previously established [27]. Social disadvantage is associated with a range of poor health behaviours, and thus obese pregnant women who come from disadvantaged backgrounds may benefit from greater intervention. Pre-conception counselling may be particularly important for this group of women. However, low socio-economic status is generally associated with poor access to health services and this, in combination with the low rates of planned pregnancies [28], suggests that community-based interventions for these women may need to be considered as a feasible alternative. The fact that women assigned to the intervention group in the RCT were more likely to be classified as *Exercisers *in late pregnancy suggests the importance of providing support for obese pregnant women to facilitate long-term health behaviour change.

Pregnancy symptoms were also associated with exercise status during pregnancy. Women who reported lower back pain at 12 and 20 weeks and nausea or vomiting at 20 and 28 weeks were less likely to be classified as *Exercisers*. These findings are consistent with other evidence suggesting that exercise in early pregnancy is related to decreased reporting of nausea and vomiting in late pregnancy [12]. However, the direction of the relationship between these pregnancy symptoms and exercise in our study was not clear. The findings may suggest that exercise helps alleviate nausea and vomiting in pregnancy. Alternatively, women may have chosen to exercise because they experienced less nausea or vomiting during their pregnancy. In this study we found physical health differences between the *Exercisers *and *Non-Exercisers*. Specifically, pre-pregnancy weight in the *Exercisers *was lower at 12 weeks gestation than in the *Non-exercisers*. The differences between the groups for back pain found in early pregnancy also support this, and are consistent with evidence suggesting that pre-pregnancy physical activity is associated with a reduced risk of back pain during pregnancy [14]. Further work is required to determine if this is a causal relationship. In our study, it is unclear whether women who have less back pain are more likely to exercise, or whether women who do exercise benefit from a reduction in back pain.

A major issue with lifestyle interventions is the assessment of physical activity. Both subjective and objective measures of physical activity have well known limitations (27). The PPAQ was very useful measure in our study - easy to complete and tailored to measure physical activity among women during pregnancy. The self-report nature of the questionnaire meant that we relied on the women to accurately recall their activity, and this may have led to an overestimation of exercise hours. Thus, our results may not generalise to other studies, particularly those relying on objective measures of exercise.

In the RCT, a number of women withdrew from the trial at different stages, limiting the data that was available for analysis over the course of the trial. A total of five women (*n *= 3, control; *n *= 2, intervention) dropped out soon after their baseline visit. Three women withdrew from the trial when they discovered they had been randomised to the control group and were disappointed with this outcome. Two women who were randomised to the exercise arm also withdrew at 12 weeks when they were diagnosed with gestational diabetes based on their baseline blood tests. Among the remaining women, those that withdrew from the study (*n *= 4, control; *n *= 5, intervention) did so because of medical or obstetric complications (e.g. miscarriage, intrauterine fetal death, sacroiliac joint instability, gestational diabetes); five women delivered before 36 weeks and thus did not have data collected at 36 weeks. The retention rates in the RCT and the reasons for non-completion have been published elsewhere [18].

The small sample size in our study limited our ability to adjust for other variables, including age and pre-pregnancy BMI. The women who remained in the study may represent a highly motivated group, which may limit the extent to which these results generalise. The results of this small pilot study suggest that it may be important to adjust for sociodemographic variables (e.g. age, education), as well as pre-pregnancy BMI in future analyses examining the correlates of exercise in pregnancy.

## Conclusions

This is the first study to identify the correlates of exercise during pregnancy in an obese population. We found that health-related variables tended to predict exercise in early pregnancy whereas sociodemographic variables were most likely to predict exercise in late pregnancy. Specifically, women who had a history of miscarriage; a lower pre-pregnancy BMI; who reported no nausea and vomiting; and who had no lower back pain, were those women who were most likely to have exercised in early pregnancy. Exercise in late pregnancy was most common among women who were better educated, and there was a trend for younger women to be classified as *Exercisers *during the third trimester of pregnancy. However, the direction of the relationship between exercise and pregnancy symptoms is unclear and requires further examination using larger samples prospectively designed to answer these questions.

The findings may be particularly useful for researchers who are designing interventions aimed at reducing or controlling weight gain in obese pregnant women. Interventions that offer greater support to women from disadvantaged backgrounds, and closer monitoring of women who report persistent nausea and vomiting or lower back pain during pregnancy may be beneficial. Providing this care and support may be an initial step towards increasing obese women's participation in exercise during pregnancy.

## Competing interests

The authors declare that they have no competing interests.

## Authors' contributions

KFF: Has been involved in design of the study, data collection, analysis and drafting the manuscripts. IJR has made substantial contributions to the statistical analysis of data and drafting of the manuscript. NMB has been involved in interpretation of the data and revising the intellectual content. HDM was involved in study design and revision of the intellectual content. LKC has been involved in the design of the study, obtained funding, provided oversight of the data collection and data analysis and revision of the intellectual content. All authors have read and approved the final manuscript.

## Authors' information

KFF: MAppSc (Research), RN, RM.

IJR: BPsycSc (Hons), PhD. Postdoctoral Research Fellow at University of Queensland and Queensland Institute of Medical Research.

NMB: BHMS, MAppSc, PhD.

HDM: MBBS (Hons), FRACP.

LKC: MBBS (Hons), FRACP, PhD.

## Pre-publication history

The pre-publication history for this paper can be accessed here:

http://www.biomedcentral.com/1471-2393/11/4/prepub
